# The Influence of Molecular Weights on Dispersion and Thermoelectric Performance of Alkoxy Side-Chain Polythiophene/Carbon Nanotube Composite Materials

**DOI:** 10.3390/polym16172444

**Published:** 2024-08-29

**Authors:** Xiaogang Chen, Shihong Chen, Dagang Wang, Yongfu Qiu, Zhongming Chen, Haixin Yang, Qing Yang, Zijian Yin, Chengjun Pan

**Affiliations:** 1 College of Chemistry and Chemical Engineering, Shenzhen University, Shenzhen 518060, China; 2110343112@email.szu.edu.cn (X.C.); chensher7@163.com (S.C.); 13720385426@163.com (H.Y.); 2110343048@email.szu.edu.cn (Q.Y.); 2110343060@email.szu.edu.cn (Z.Y.); 2 School of Environment and Civil Engineering, Dongguan University of Technology, Dongguan 523808, China; qiuyf@dgut.edu.com (Y.Q.); zmchen@dgut.edu.cn (Z.C.)

**Keywords:** organic thermoelectric materials, composite thermoelectric device, alkoxy side chain

## Abstract

In the development of wearable electronic devices, the composite modification of conductive polymers and single-walled carbon nanotubes (SWCNTs) has become a burgeoning research area. This study presents the synthesis of a novel polythiophene derivative, poly(3-alkoxythiophene) (P3(TEG)T), with alkoxy side chains. Different molecular weight variants of P3(TEG)T (P1–P4) were prepared and combined with SWCNTs to form composite materials. Density functional theory (DFT) calculations revealed a reduced bandgap for P3(TEG)T. Raman spectroscopy demonstrated π-π interactions between P3(TEG)T and SWCNTs, facilitating the dispersion of single-walled carbon nanotubes and the formation of a continuous conductive network. Among the composite films, P4/SWCNTs-0.9 exhibited the highest thermoelectric performance, with a power factor (PF) value of 449.50 μW m^−1^ K^−2^. The fabricated flexible thermoelectric device achieved an output power of 3976.92 nW at 50 K, with a tensile strength of 59.34 MPa for P4/SWCNTs. Our findings highlight the strong interfacial interactions between P3(TEG)T and SWCNTs in the composite material, providing an effective charge transfer pathway. Furthermore, an improvement in the tensile performance was observed with an increase in the molecular weight of the polymer used in the composite, offering a viable platform for the development of high-performance flexible organic thermoelectric materials.

## 1. Introduction

The popularity of wearable electronic devices has soared in recent years due to their novelty, lightweight design, and comfortable nature. However, the incompatibility and rigidity of traditional power supply systems have left many individuals seeking alternative solutions. Flexible and convenient self-charging energy systems have emerged as ideal solutions to meet the power demands of wearable electronic devices. Various types of self-powered devices have been successfully developed, including triboelectric nanogenerators (TENG) [1], piezoelectric nanogenerators (PENG) [2], photovoltaic devices [3], and thermoelectric devices [4]. Among these, thermoelectric materials utilizing the Seebeck effect have shown promise in generating electrical power. These thermoelectric generators can provide power to electronic devices without the need for external power sources or other media. These energy-harvesting devices can be integrated with energy storage systems such as batteries, further enhancing their integration into flexible and wearable electronic devices [5,6].

Research on thermoelectric materials can be categorized into two major classes: inorganic thermoelectric materials and organic thermoelectric materials [7]. At present, within the realm of traditional inorganic thermoelectric materials, the emphasis is primarily placed on high-performance inorganic semiconductor materials, such as bismuth telluride (Bi_2_Te_3_) [8,9] and antimony telluride (Sb_2_Te_3_) [10] alloys. Additionally, compounds of inorganic thermoelectric materials, like copper selenide (Cu_2_Se) [11,12] and lead telluride (PbTe) [13], have achieved a dimensionless figure of merit (ZT) exceeding 2.0. At present, the widespread commercialization of inorganic thermoelectric materials is limited due to their toxicity, high cost, and poor mechanical properties. The exploration of thermoelectric properties in a diverse range of p-type conjugated organic materials has become a focal point, stemming from the discovery of their low thermal conductivity [14,15]. Upon internal structural analysis, it is evident that organic materials possess a lower thermal conductivity compared to their inorganic counterparts due to the presence of weak van der Waals forces and disordered microstructures within the polymers [16]. In comparison to inorganic thermoelectric materials, organic thermoelectric materials offer advantages such as ease of processing, low thermal conductivity, cost-effectiveness, pliability, excellent bendability, flexibility, and lightweight properties. Simultaneously, there is a growing demand for novel intelligent devices, such as flexible wearable electronics and artificial intelligent skin, where flexible thermoelectric materials can harness temperature differentials between the human body and the environment to generate power without the need for battery devices. Furthermore, their flexible mechanical properties can meet the daily usage requirements of flexible devices, rendering the research and application prospects of flexible thermoelectric materials highly promising [17,18,19,20]. However, the immense advantage of low thermal conductivity in organic thermoelectric materials still lacks effective competitiveness in commercial applications due to their low electrical conductivity and modest Seebeck coefficient. Nevertheless, the complementary nature of their advantages can be harnessed through strategies such as doping and the fabrication of composite materials, thereby improving and optimizing the performance of organic thermoelectric materials. Composite materials consisting of carbon nanotubes and polymers have progressively matured, with the related research indicating that multistage mechanical activation can facilitate their excellent integration, leading to enhanced thermoelectric parameters. Furthermore, composite materials comprising different polymers and carbon nanotubes have demonstrated commendable improvements in their thermoelectric performance. Combining conductive carbon nanotubes with polymers has opened up avenues for researchers to explore beyond the thermoelectric properties, such as the mechanical performance and various other aspects, thereby extending the potential applications of these composites [14,21,22,23,24]. The main challenge in the development of such materials lies in striking a balance between a high thermoelectric performance and flexibility under stretching conditions.

Viscoelastic conjugated polymers, owing to the influence of their structure, exhibit outstanding electrical properties and flexibility. They hold enormous potential in the research of flexible thermoelectric materials. In recent years, researchers have made significant progress in the development of composite materials combining conjugated polymers with carbon nanotubes [25,26,27]. In particular, fruitful achievements have been obtained in the study of polythiophene derivatives and single-walled carbon nanotubes [28,29], addressing some of the challenges faced by such composite materials. For instance, Lin et al. [30] demonstrated that branched alkylthio side chains can enhance the dispersion of carbon nanotubes within polythiophene derivatives and yield a promising thermoelectric performance. Hao et al. [31] showed that the addition of polar side chains can also modify and enhance the thermoelectric properties of poly(3-substituted thiophene)/single-walled carbon nanotube composites. The addition of side chains and the proportion of the carbon nanotubes can indeed impact the thermoelectric performance of composite materials. However, research on the thermoelectric performance of polythiophene combined with single-walled carbon nanotubes at different molecular weights remains limited. There are few reports on whether varying the molecular weight of the added polymer affects both the thermoelectric and mechanical properties of the composite materials. Building upon the existing research, our study aims to create new side-chain polythiophenes that can enhance their thermoelectric performance. We will investigate the effects of different molecular weights of the added polymer on aspects such as the thermoelectric performance and mechanical properties, with the optimal composite ratio of polythiophene and carbon nanotubes as a basis for our investigation.

In this study, different molecular weight variants of polythiophene—namely poly(3-alkoxythiophene) with alkoxy side chains (P3(TEG)T) (P1–P4)—were synthesized and combined with single-walled carbon nanotubes (SWCNTs) to fabricate composite thermoelectric materials and flexible thin-film thermoelectric devices. The influence of the interaction between the polymers with different molecular weights and the SWCNTs on the thermoelectric properties was systematically investigated. Among the composite films, P4/SWCNTs-0.9 exhibited the highest thermoelectric performance, with a power factor (PF) value of 449.50 μW m^−1^ K^−2^. The fabricated flexible thermoelectric device achieved an output power of 3976.92 nW at 50 K, with a tensile strength of 59.34 MPa for P4/SWCNTs. Our study demonstrates a strong interfacial interaction between the P3(TEG)T and SWCNTs in the composite material, providing a charge transfer pathway. Furthermore, we observed that an increase in the molecular weight of the polymer used in the composite led to an improved tensile performance. These findings offer a new perspective for the development of high-performance flexible organic thermoelectric materials.

## 2. Experimental

### 2.1. Materials

The reagents and solvents were obtained from Sigma-Aldrich, Tokyo Chemical Industry (TCI), and Zhengzhou Alpha Chemical Co., Ltd. (Zhengzhou, China). Thermoelectric grade single-walled carbon nanotubes (purity of 80%) were obtained from Xianfeng Nanomaterials for this study.

### 2.2. Fabrication of Polymer/SWCNTs Composite Film

The composite films produced in this study are p-type organic composite films prepared using the drop-casting method. Taking the preparation of the P3ODT/SWCNTs composite film as an example, the specific operational steps are as follows:

1. Ultrasonic dispersion of single-walled carbon nanotubes: Weigh 40 mg of 5–10 μm long single-walled carbon nanotubes into a 100 mL round-bottom flask. Prepare a 1 mg mL^−1^ dispersion in anhydrous chlorobenzene solution. Then, ultrasonicate the mixture in an ultrasonic cleaner for 5 h.
2. Preparation of different ratios of composite solution: Prepare composite solutions with mass ratios of 1:9, 3:7, 5:5, 7:3, and 9:1 for P3ODT/SWCNTs. Label each composite solution accordingly. Subsequently, place the composite solutions in the ultrasonic cleaner and sonicate for 2 h to ensure uniform dispersion.
3. Preparation of P3ODT/SWCNTs composite film using the drop-casting method: First, clean a 1 cm × 1 cm glass slide (1 mm thickness) sequentially with dichloromethane, deionized water, and acetone through ultrasonication for half an hour. After drying the glass slide, slowly drop-cast 120 μL of the different mass ratio composite solutions onto the glass slide using a pipette. Allow the chlorobenzene solvent to evaporate naturally at room temperature for half an hour to obtain composite films with different mass ratios of P3ODT/SWCNTs. The labeling of the film samples is based on the percentage of SWCNTs present. For example, if SWCNTs account for 50% of the composite film, it is labeled as P3ODT/SWCNTs-0.5.

### 2.3. Manufacture and Measurement of a Flexible Thermoelectric Device

The operational steps for preparing the polymer/SWCNTs composite thermoelectric devices in this study are as follows:

1. Prepare the required composite solution.
2. Cut the flexible polyimide (PI) film into rectangular strips measuring 1 cm × 4 cm. Sequentially ultrasonic with dichloromethane, deionized water, and acetone for half an hour; dry after cleaning.
3. Pipette 300 μL of the prepared composite solution onto the PI film. Allow it to naturally evaporate at room temperature.
4. Take the 1 cm × 4 cm composite films and adhere them onto an 8 cm × 24 cm PI film using double-sided tape with a 1 cm spacing between each composite film. Then, connect the composite films and the copper foil tape at the intersections using conductive silver paste to ensure good electrical contact and to reduce contact resistance.

The prepared thermoelectric devices were tested on a Keithley 4200A-SCS measurement platform. A temperature control heating stage was used to create a temperature gradient across the thermoelectric device.

## 3. Results and Discussion

### 3.1. Synthesis and Evaluation of Polymers

**Figure 1:**
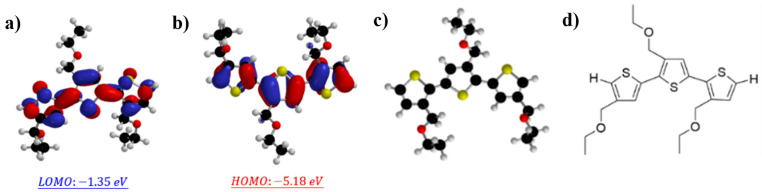
(**a**,**b**) DFT-calculated HOMO and LUMO energy levels of polymer P3(TEG)T dimer and (**c**,**d**) plane structure.

**Figure 2:**
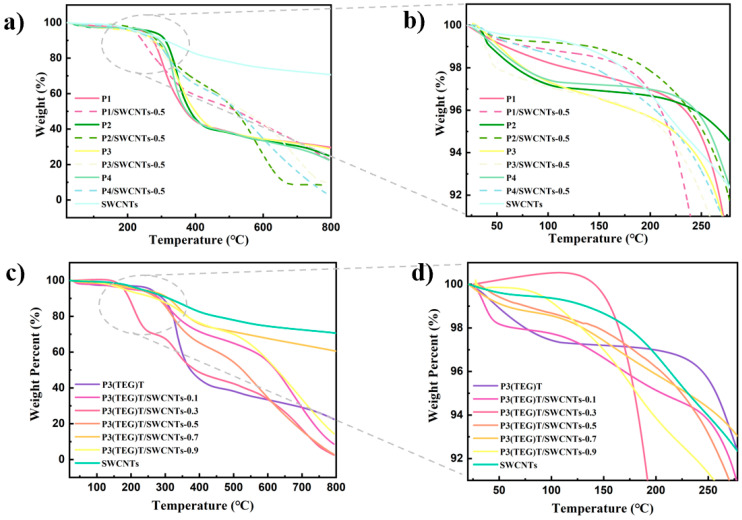
TGA curve of (**a**,**b**) polymer P3(TEG)T (P4), SWCNTs and composite P3(TEG)T/SWCNTs, (**c**,**d**) TGA curve of polymer P1–P4, SWCNTs and composites.

The various polymers studied in this paper were synthesized with different molecular weights and branched alkoxy side chains using the controllability of Grignard exchange reactions. The synthesis of poly(3-alkoxythiophene) with branched alkoxy side chains, namely P3(TEG)T, with different molecular weights was achieved by controlling the ratio of monomer to catalyst, as described in the experimental section [28]. The chemical structures of the synthesized P3(TEG)T are shown in Figure 1. Therefore, in this study, P1–P4 refers to P3(TEG)T with different molecular weights. The interaction between the P3(TEG)T and SWCNTs exhibited strong π-π adsorption, improving the dispersion of single-walled carbon nanotubes in organic solvents. Figure S1 provides a representative 1H NMR spectrum of polymer P3(TEG)T using deuterated chloroform as the solvent. Different molecular weights of alkoxyl side-chain polythiophene P3(TEG)T were characterized for their molecular weight and dispersity using gel permeation chromatography (GPC). The synthesized alkoxyl side-chain polythiophene was dissolved in chromatography-grade tetrahydrofuran (THF) to prepare a 2 mg mL^−1^ solution, which was then filtered through a membrane filter before subjecting it to GPC analysis. The GPC chromatograms for alkoxyl side-chain polythiophene P3(TEG)T with different molecular weights are shown in Figure S7. The GPC curve provides a clear representation of the molecular weight distribution of polythiophene P3(TEG)T. Combined with the polydispersity index (PDI), number-average molecular weight (Mn), weight-average molecular weight (Mw), and degree of polymerization (DPn) data presented in Table 1, it is evident that different molecular weights of P1–P4 were obtained by controlling the monomer-to-catalyst equivalent ratio during the synthesis process.. As shown in Figure 2, the high thermal stability of the studied polymers was confirmed through thermogravimetric analysis (TGA), indicating a 5% weight loss temperature (Td5% s) in the range of 183.4–269.36 °C.

**Figure 3:**
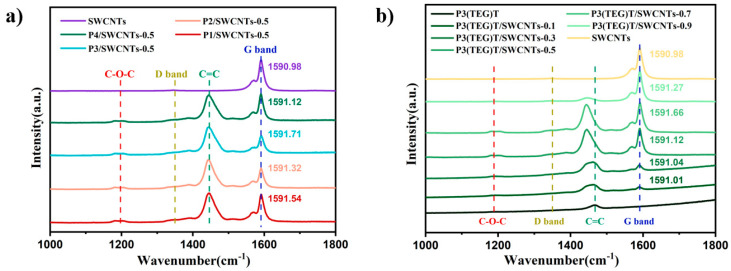
Raman spectra of composite films (**a**) P3(TEG)T/SWCNTs and (**b**) P1–P4/SWCNTs-0.5.

The energy levels of the P3(TEG)T polymer were studied through density functional theory (DFT) calculations, as shown in Figure 1. The polymer was simplified by replacing the long alkoxy side chains of P3(TEG)T with ethoxy methyl groups, and trimer units were used for B3LYP/6-31G* model calculations. It can be observed that the HOMO and LUMO energy levels of the trimer are delocalized over the entire thiophene backbone, indicating a significant π-conjugated structure of the trimer. The calculated HOMO and LUMO energy levels of P3(TEG)T are −5.18 eV and −1.35 eV, respectively, resulting in a calculated bandgap of 3.83 eV. Figure 3 presents a relative energy diagram of the interface between the polythiophene and single-walled carbon nanotubes (SWCNTs). All of the polythiophene derivatives exhibit HOMO levels sufficiently similar to that of the pristine SWCNTs (−5.10 eV), making the interaction between them possible [26].

### 3.2. Spectroscopic Properties of Composites

Utilizing Laser Coherent Raman Spectroscopy within the range of 500–2000 cm^−1^, we conducted tests on synthesized polymer P3(TEG)T, prepared P3(TEG)T/SWCNTs composite films, as well as composite films of different molecular weights of polymers P1–P4 with SWCNTs. The obtained Raman spectra are presented in Figure 3, where P3(TEG)T represents polymer P4 in Figure 3a.

In Figure 3, the absorption peak around 1470 cm^−1^ corresponds to the symmetric stretching vibration of the C_α_=C_β_ double bond in the main thiophene groups of polymer P3(TEG)T. The absorption peak at approximately 1185 cm^−1^ is attributed to the stretching vibration of the C-O-C bonds in the polymer [32]. The absorption peaks around 1590 cm^−1^ and 1570 cm^−1^ correspond to the G-band of SWCNTs, denoted as G^+^ and G^−^, respectively, arising from the E2g mode of the carbon atoms in the hexagonal lattice and sp^2^ hybridization [33,34]. This indicates a significant presence of sp^2^-hybridized carbon atoms, facilitating charge transport. There is no noticeable D-band absorption peak around 1350 cm^−1^, indicating the absence of defects in the SWCNT material [35]. Moreover, the composite films show no apparent structural defects, promoting charge transfer and contributing to an improved thermoelectric performance.

**Figure 4:**
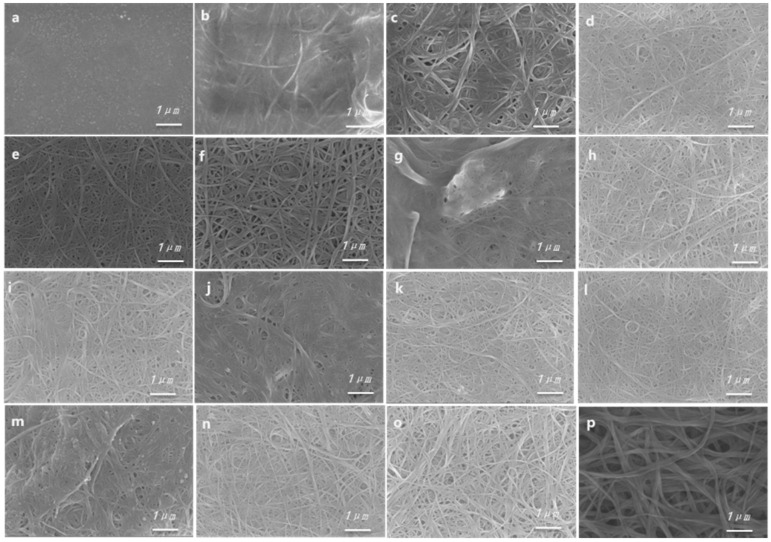
SEM images of (**a**) the P3(TEG)T (P4) film, the P4/SWCNTs composite films with various mass ratio (**b**) 9:1; (**c**) 7:3; (**d**) 5:5; (**e**) 3:7; (**f**) 1:9, the P3/SWCNTs composite films with various mass ratio (**g**) 7:3; (**h**) 5:5; (**i**) 3:7, the P2/SWCNTs composite films with various mass ratio: SWCNTs (**j**) 7:3; (**k**) 5:5; (**l**) 3:7, the P1/SWCNTs composite films with various mass ratio (**m**) 7:3; (**n**) 5:5; (**o**) 3:7 and (**p**) SWCNTs film.

In Figure 3a, as the content of SWCNTs increases in the P3(TEG)T/SWCNTs composite film, the symmetric stretching vibration peak of the C_α_=C_β_ double bond exhibits a pronounced redshift, accompanied by changes in the peak intensity. The stretching peak of C-O-C appears, and the intensity of the G-band increases. These observations suggest a strong π-π interface interaction between the P3(TEG)T and SWCNTs, as well as an ordered arrangement of the alkoxyl side chains, which enhances the charge transport. In Figure 3b, the Raman spectra of the P3(TEG)T/SWCNTs composite films with different molecular weights show no significant differences. The G-band intensity decreases after the addition of the polymer, indicating a well-formed composite film with sufficient polymer-SWCNTs interaction through the π-π interface. Additionally, in Figure 4a,b, a redshift of the G-band compared to the SWCNTs is observed in the composite materials, indicating the transfer of Fermi level electrons from the SWCNTs to the LUMO level of the polymer, resulting in effective p-type doping [35].

### 3.3. Morphological Properties of Composites

To gain a deeper understanding of the nanoscale structure of the composite materials, we employed scanning electron microscopy (SEM) to investigate the surface morphology of the polymer P3(TEG)T (P4), SWCNTs, and composite films P1–P4/SWCNTs. As shown in Figure 4, Figure 4a depicts the surface morphology of the P3(TEG)T (P4) film, while Figure 4b–f present the SEM images of the P4/SWCNTs composite films with increasing proportions of SWCNTs. Similarly, Figure 4g–o display the SEM images of the P3/SWCNTs, P2/SWCNTs, and P1/SWCNTs composite films, respectively. Figure 4p shows the surface morphology of the pure SWCNTs film.

In Figure 4a, the surface of the polymer P3(TEG)T film appears relatively smooth, without any visible aggregation. Figure 4p demonstrates a uniform distribution of SWCNTs without aggregation, indicating good dispersion. In Figure 4b–o, with an increasing proportion of SWCNTs in the composite films, a uniform distribution of the SWCNTs is observed, and a network-like fibrous structure of the SWCNTs emerges within the composite films. This is attributed to the uniform encapsulation of the polymer on the surface of the individual carbon nanotubes, leading to strong interactions between the two components in the composite material and facilitating charge carrier transport [31]. Notably, the fiber bundles increase in size and exhibit increased interconnection between different bundles, forming a conductive network favorable for charge transfer. This effectively enhances the electrical conductivity of the composite film, thereby contributing to the improvement of its thermoelectric properties. The effective and stable connection between the conjugated polymer materials and the surface of the single-walled carbon nanotubes enables rapid electron transport, potentially facilitating the development of high-performance thermoelectric films.

### 3.4. Thermoelectric Properties of Composite Thin Films

**Figure 5:**
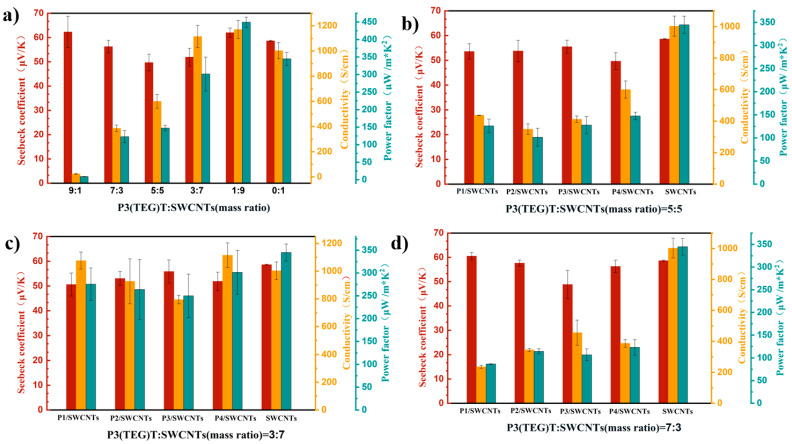
Thermoelectric properties of polymer P3(TEG)T/SWCNTs composite (**a**), P1–P4/ SWCNTs-0.3 (**b**), P1–P4/ SWCNTs-0.5 (**c**), P1–P4/ SWCNTs-0.7 (**d**).

The thermoelectric performance of the P3(TEG)T/SWCNTs composite film, where P3(TEG)T refers to polymer P4, varies with the content of SWCNTs at room temperature. Figure 5a illustrates the thermoelectric performance of the P3(TEG)T/SWCNTs composite film. The thermoelectric performance graphs of the P1–P4/SWCNTs composite films, with SWCNTs proportions of 30%, 50%, and 70%, are presented in Figure 5b,c,d, respectively. The Seebeck coefficients of the P3(TEG)T/SWCNTs composite films are all positive, indicating their classification as p-type thermoelectric materials, with charge carriers being conducted through the holes. As the proportion of SWCNTs increases, the Seebeck coefficient of P3(TEG)T/SWCNTs initially decreases and then increases. Among them, the Seebeck coefficient of P3(TEG)T/SWCNTs-0.9 reaches its maximum value of 62.01 μV K^−1^, which is higher than that of pure SWCNTs. The electrical conductivity of the composite films increases with the addition of SWCNTs. This is because the introduction of SWCNT bundles provides more efficient pathways for rapid electron transfer. The electrical conductivity of P3(TEG)T/SWCNTs-0.7 and P3(TEG)T/SWCNTs-0.9 is higher than that of pure SWCNTs, measuring 1115.33 S cm^−1^ and 1170.90 S cm^−1^, respectively. Furthermore, the power factor of the composite films also increases with the incorporation of SWCNTs. The highest power factor is achieved in P3(TEG)T/SWCNTs-0.9, measuring 449.50 μW m^−1^ K^−2^, surpassing that of pure SWCNTs. This is attributed to the strong π-π interactions formed between the P3(TEG)T and SWCNT bundles, facilitating charge transfer and improving the thermoelectric performance of the composite film [36].

In Figure 5b–d, the P1/SWCNTs-0.3 composite film exhibits the highest Seebeck coefficient of 60.56 μV K^−1^ when the proportion of SWCNTs is 30%. The highest electrical conductivity is observed in P3/SWCNTs-0.3, measuring 455.63 S cm^−1^, and the maximum power factor is achieved in P4/SWCNTs-0.3, measuring 122.89 μW m^−1^ K^−2^. When the proportion of SWCNTs is 50%, the P3/SWCNTs-0.5 composite film exhibits the highest Seebeck coefficient of 55.56 μV K^−1^. The P4/SWCNTs-0.5 composite film demonstrates the highest electrical conductivity and power factor, measuring 600.41 S cm^−1^ and 147.51 μW m^−1^ K^−2^, respectively. In the case of a 3:7 ratio of polymer to SWCNTs, the P3/SWCNTs-0.7 composite film exhibits the highest Seebeck coefficient of 55.88 μV K^−1^. The P4/SWCNTs-0.7 composite film demonstrates the highest electrical conductivity and power factor, measuring 1115.33 S cm^−1^ and 301.67 μW m^−1^ K^−2^, respectively.

The occurrence of a high Seebeck coefficient but a relatively low power factor can be attributed to the trade-off between the increase in electrical conductivity and the decrease in the Seebeck coefficient in the composite material. With an increase in the molecular weight of the polymer, stronger π-π interactions occur between P4 and the SWCNTs, leading to a structure that favors charge transfer and enhances the electrical conductivity, and gradually becoming the dominant factor influencing the power factor (PF) value.

### 3.5. Evaluation of Flexible P3(TEG)/SWCNTs Composite Thermoelectric Device

This study aims to investigate the thermoelectric conversion performance of a flexible P-type thermoelectric device consisting of 10 thermoelectric elements fabricated using a composite film of P3(TEG)T/SWCNTs. The composite film prepared with polymer P4 was selected for testing purposes; namely, P3(TEG)T/SWCNTs-0.9.

**Figure 6:**
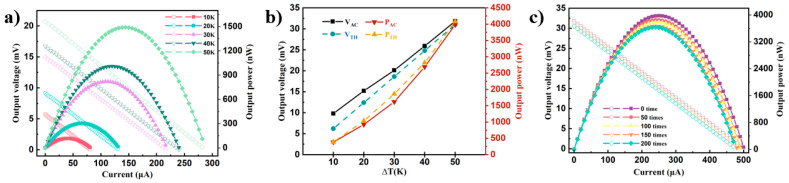
(**a**) Output power-current curve (solid circles) and open circuit voltage-current curve (open circles) of composite film devicesP3(TEG)T/SWCNTs under different temperature differences (ΔT), (**b**) Curve of P3(TEG)T/SWCNTs with temperature difference (ΔT), theoretical open circuit voltage (V_TH_), actual open circuit voltage (V_AC_), theoretical output power (P_TH_) and actual output power (P_AC_), (**c**) Output power-current curve (solid circles)and open-circuit voltage-current curve (open circles) of P3(TEG)T/SWCNTs composite thin film devices after bending at 50 K.

Figure 6a illustrates the power-current and open-circuit voltage-current curves of P3(TEG)T/SWCNTs under different temperature gradients. At the same temperature difference, the output voltage decreases with the increasing current. The output power of the P3(TEG)T/SWCNTs device continuously increases with larger temperature differences. At a temperature difference of 50 K, the maximum open-circuit voltage is 31.83 mV, and the maximum output power is 3976.92 nW. This can be attributed to the stronger π-π adsorption between the P3(TEG)T polymer, which possesses alkyl side chains, and the SWCNTs, which facilitates the movement of thermal charge carriers.

Figure 6b presents the theoretical open-circuit voltage (VTH), actual open-circuit voltage (VAC), theoretical output power (PTH), and actual output power (PAC) of the P3(TEG)T/SWCNTs thermoelectric device under different temperature differences. These values are calculated using Equations (1) and (2):

(1) $$V_{TH}=n\left(S_{p}+S_{n}\right)\Delta T$$

(2) $$P_{TH}=\frac{V_{TH}}{4R_{i}}$$

In the equations, n, S_p_, S_n_, and R_i_ represent the number of P-N thermoelectric elements, the Seebeck coefficients of P- and N-type materials, and the internal resistance of the device, respectively. It is observed that *V_TH_*, VAC, *P_TH_*, and PAC all increase with larger temperature differences. The slightly higher VAC compared to *V_TH_* may be due to measurement deviations when determining the temperature difference between the two ends. Overall, the trends of VAC and VTH, as well as PTH and PAC, follow the theoretical expectations, adhering to the device’s principles.

Figure 6c displays the power-current and open-circuit voltage-current curves of P3(TEG)T/SWCNTs under a temperature difference of 50 K after folding. It is observed that both the output voltage and output power slightly decrease after bending. The maximum output power decreases by 343.92 nW, indicating the excellent flexibility of the thermoelectric device in terms of its resistance to bending.

**Figure 7:**
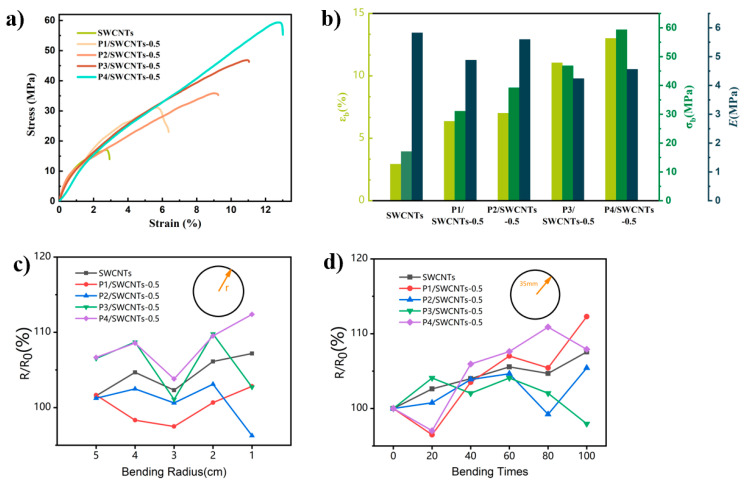
Tensile stress-strain diagram of (**a**) composite film P3(TEG)T/SWCNTs-0.5, (**b**) Tensile modulus of elasticity, fracture stress and elongation at break, (**c**) resistance variation diagram of bending times and (**d**) resistance variation diagram of different bending radius.

Mechanical tensile tests and bending tests were conducted to study the mechanical properties of the P1–P4/SWCNTs-0.5 and SWCNTs composite films. Figure 7 illustrates the stress-strain curves for the mechanical tensile testing and the graphs of the tensile elastic modulus, fracture stress, and fracture elongation for the P1–P4/SWCNTs-0.5composite film. Figure 7a,b show the mechanical tensile stress-strain curves for the SWCNTs and the P1–P4/SWCNTs-0.5composite film, respectively. It is observed that both composite films exhibit higher mechanical tensile performances compared to the pure carbon nanotubes. This is attributed to the strong interfacial interaction formed between different molecular weight polymers, P1–P4, and SWCNTs during the composite process, which enhances their effective stress. Moreover, it is found that the mechanical tensile performance improves with the increasing molecular weight of the polymer used in the composite material. Specifically, the tensile strength of P4/SWCNTs reaches 59.34 MPa, indicating a stronger interfacial force between P4 and the SWCNTs due to its higher molecular weight.

Figure 7c demonstrates the resistance variation of the composite film under different bending frequencies at a curvature radius of 35 mm. Figure 7d shows the resistance variation of the composite film after 50 bending cycles at different curvature radii. It is observed that the resistance variation rate remains within the range of 97% to 112.5% for the different composite films and SWCNTs under the same bending curvature radius, indicating relative stability. Additionally, the resistance variation before and after 50 bending cycles at different curvature radii remains small, ranging between 98% and 112.5%. These findings suggest that the P1–P4/SWCNTs-0.5 composite film possesses excellent flexibility and durability, which is beneficial for the application and research of flexible devices. Furthermore, this composite film enables the enhancement of the thermoelectric performance of nanocomposite materials to a competitive level.

## 4. Conclusions

In order to investigate the effect of the molecular weight on the thermoelectric performance of polythiophene (PT)/single-walled carbon nanotubes (SWCNTs) composite materials, a novel polythiophene derivative with alkoxyl side chains—poly(3-alkoxy straight chain thiophene) (P3(TEG)T)—was designed and synthesized. Composite materials composed of different molecular weight P3(TEG)T (P1–P4) and SWCNTs were also synthesized. Raman spectroscopy and SEM morphology analysis revealed strong π-π interface interactions between P3(TEG)T and the SWCNTs, and the introduction of alkoxyl straight chains improved the dispersibility of polythiophene in the carbon nanotubes. A flexible thermoelectric transfer material was constructed using the p-type P3(TEG)T/SWCNTs nanocomposite film, and the results showed that the composite film with the highest molecular weight, P4/SWCNTs-0.9, exhibited an excellent thermoelectric performance with a power factor (PF) of 449.50 μW m^−1^ K^−2^. The flexible thermoelectric device produced an output power of 3976.92 nW at 50 K, and the tensile strength of P4/SWCNTs reached 59.34 MPa. The composite of high molecular weight alkoxyl side-chain polythiophene P3(TEG)T with SWCNTs enhances the tensile strength of the composite material. Simultaneously, the highest thermoelectric performance was observed in the composite films containing high molecular weight polymers. Therefore, this work not only demonstrates the improvement of the thermoelectric and tensile properties through the optimization of the molecular weight, but also further explores the application of PT/SWCNTs composite materials in wearable devices.

## Figures and Tables

**Table 1:** GPC parameters of polythiophene P1–P4.

Polymer	*M_n_* (kDa)	*M_w_* (kDa)	PDI	DP_n_
**P1**	18.4	28.4	1.53	71
**P2**	22.9	31.7	1.38	89
**P3**	27.3	43.4	1.37	105
**P4**	29.8	49.3	1.65	115

## Data Availability

Not applicable.

## Supplementary Materials

The following supporting information can be downloaded at: https://www.mdpi.com/article/10.3390/polym16172444/s1. Refs. [37,38,39,40,41,42] are cited in the supplementary materials.
