# Supplementary material for: The Influence of Molecular Weights on Dispersion and Thermoelectric Performance of Alkoxy Side-Chain Polythiophene/Carbon Nanotube Composite Materials
Source: Polymers (Basel). 2024 Aug 29;16(17):2444. doi: 10.3390/polym16172444 (PMC11397576; doi:10.3390/polym16172444)
Supplement: Supplementary file 1 [file polymers-16-02444-s001.zip › polymers-2612877-supplementary.pdf]

Supplementary Materials

The Influence of Molecular Weights on Dispersion and Thermoelectric Performance of Alkoxy Side-Chain Polythiophene/Carbon Nanotube Composite Materials

Xiaogang Chen [1,†], Shihong Chen [1,†], Dagang Wang [1], Yongfu Qiu [2], Zhongming Chen [2], Haixin Yang [1], Qing Yang [1], Zijian Yin [1] and Chengjun Pan [1,*]

[1] College of Chemistry and Chemical Engineering, Shenzhen University, Shenzhen 518060, China; 2110343112@email.szu.edu.cn (X.C.); chensher7@163.com (S.C.); wangdagang@szu.edu.cn (D.W.); 13720385426@163.com (H.Y.); 2110343048@email.szu.edu.cn (Q.Y.); 2110343060@email.szu.edu.cn (Z.Y.)

[2] School of Environment and Civil Engineering, Dongguan University of Technology, Guangdong 523808, China; qiuyf@dgut.edu.com (Y.Q.); zmchen@dgut.edu.cn (Z.C.)

* Correspondence: pancj@szu.edu.cn

† These authors contributed equally to this work.

Instruments. ^{1}H nuclear magnetic resonance (^{1}H NMR) spectra for polymer characterization were recorded on a Nuclear Magnetic Resonance Spectrometer Bruker（400MHz- 600MHz）in $C_2D_2Cl_4$ The number average and weight average molecular weights (M_n and M_w) and molar-mass dispersity ($Đ_M$) were measured via Gel permeation chromatography（GPC）， the tested samples were prepared in chromatographically pure tetrahydrofuran as a 2 mg/mL solution for testing. Thermal Stability Analysis (TSA) is the measurement of the thermal stability of a sample by measuring the chemical or physical changes of the sample in the heated state, where the temperature at 5% weight loss is defined as the thermal decomposition temperature of the material. The instrument used was a TGA-55 with a sample size of 10 mg, set to ramp up at 10°C·min^{-1} in the range of 25°C-800°C, and tested in a nitrogen-protected environment. Raman spectra were collected with a RENIDHAW in Via (514 nm). Fluorescence spectra were measured using a Jobin Yvon Fluorolog-3 Spectrofluorometer. Scanning electron microscope (SEM) studies was conducted with a SU-70. The tensile test apparatus is CMT4204 microcomputer universal material testing machine, the test sample is prepared into dumbbell national standard type 2 through PTFE mold, where the narrow length is 25 mm, the narrow width is 4 mm, and the thickness is 150 μm. the instrument tensile program is set to 0.5 mm/min, stretching until the end of fracture, bending test, the composite material is cut into 2×4 cm^2 rectangles, record The resistance of different bending radii and bending times were recorded to finally obtain the mechanical properties related to the composites. The thin film thermoelectric properties were tested using the MRS-3 equipment, and the temperature difference was applied to the film to measure the electric potential and obtain the desired Seebeck coefficient S and conductivity σ, where the conductivity σ was calculated from the resistance between the two probes on the sample. The composite solution was dried into a film at room temperature after drop coating before testing, and the film thermoelectric properties were tested at 298 K. The power factor PF value of the composite was finally calculated, where the film thickness was tested by ET-4000M two-dimensional microfabrication tester. The equipment used for the performance testing of thermoelectric devices is Keithley 4200A-SCS test platform. Before testing, the composite material is prepared into a 1×4 cm^2 size film, and a thermoelectric test device (TEG) with 10 thermocouples is connected to the PI film with copper glue. During the test, one end of the

TEG test device is heated and one end is in the air, and the temperature difference between the two ends is observed in real time by contact thermocouple thermometer and infrared imager, and the temperature difference between the two ends is created by adjusting the temperature of the temperature regulating heating table, so as to obtain the open circuit voltage and output power of the thermoelectric device under different temperature differences.

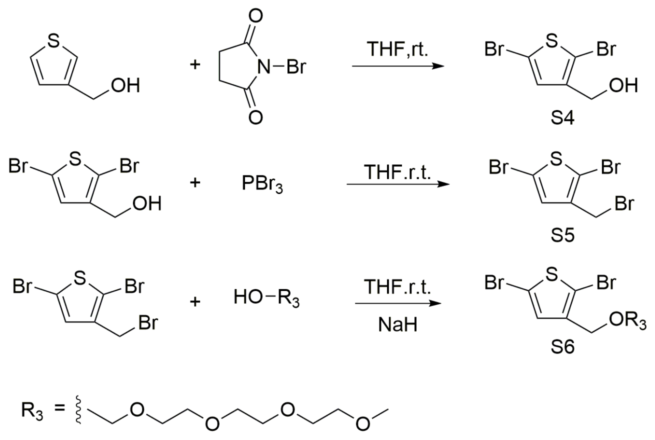

Figure S1 Synthetic routes of intermediate compounds and monomer

1）Synthesis of intermediate 2,5-dibromo-3-thiophenemethanol S4.

As shown in Figure 4-1, the synthesis of intermediate 2,5-dibromo-3-thiophenemethanol S4 used a bromination reaction, and the specific relevant experimental operations were as follows:

A 250 mL two-neck flask H was connected to a vent valve and sealed, and the flask was pumped three times to remove water and oxygen and ensure that the flask was filled with nitrogen. Weigh 10 g of 3-thiophene methanol (1 eq, 54.75 mmol) and inject it into flask H. Inject 50 mL of super-dry tetrahydrofuran under nitrogen to dissolve it; weigh 2.69 g of N-bromosuccinimide (2.2 eq, 120.45 mmol) in another 100 mL flask I and add 60 mL of super-

dry tetrahydrofuran was added under nitrogen to form a suspension, and the suspension was slowly injected into two flasks H with a syringe in batches under an ice-water bath, maintained at 0 °C for half an hour and then warmed to room temperature to react overnight. After the reaction was finished, the organic phase was extracted with hexane and 10% aqueous sodium thiosulfate, dried with anhydrous magnesium sulfate and filtered, and the crude product was obtained by distillation under reduced pressure. The crude product was purified by silica gel column chromatography, in which the eluent was ethyl acetate: hexane = 1:4. The final product Intermediate S4 was obtained: 12.67 g of pale yellow transparent oily liquid in 86% yield. ^{1}HNMR(500MHz, C_2D_6OS) δ/ppm 7.15(s，1H),5.33(t，J = 5.7Hz，1H),4.33（d，J = 5.5Hz，2H）.

2) Synthesis of intermediate 2,5-dibromo-3-methylenebromothiophene S5

The 250 mL two-necked flask J was connected to a venting valve and evacuated-vacuumed with nitrogen gas for three times to remove oxygen and water. Then 10 g of 2,5-dibromo-3-thiophenemethanol S3 (1 eq, 36.75 mmol) was weighed by syringe and injected into the two-necked flask J. 150 mL of super-dry dichloromethane solution was extracted and injected under nitrogen atmosphere, and S4 was dissolved well by stirring, and 10.95 g of phosphorus tribromide (1.1 eq, 40.43 mmol) was weighed by syringe and slowly added dropwise to the two-necked flask J under ice and water bath. After half an hour of reaction, the ice water bath was withdrawn and the reaction was carried out for 5 hours at room temperature and protected from light. After the reaction, the organic phase was extracted with dichloromethane and 10% aqueous sodium bicarbonate, and then washed with distilled water for several times, and then dried with anhydrous magnesium sulfate and filtered, and the crude product was obtained by distillation under reduced pressure. The crude product was purified by silica gel column chromatography, in which the eluent was dichloromethane : hexane = 3:2. The intermediate product S5 was finally obtained: 11.2 g of pale yellow transparent oily liquid in 88%

yield.^{1}HNMR(500MHz, C_2D_6OS) δ/ppm 7.00(s, 1H),4.36(s, 2H),4.33（d, J = 5.5Hz, 2H）.

3) Synthesis of monomeric 2,5-dibromo-3-alkoxy straight chain thiophene S6

In a 50 mL two-bottom flask K, 0.32 g of sodium hydride (60% in oil) (1.5 eq, 8.07 mmol) was weighed, and 3.5 mL of super-dry tetrahydrofuran solution was injected with a syringe after three air changes to make a suspension; an additional 1.8 g of triethylene glycol monomethyl ether (1 eq, 5.38 mmol) was weighed in another dry 50 mL two-bottom flask L, and 4.5 mL of super-dry tetrahydrofuran solution was added to the two-bottom flask L, also after three air changes. mmol), also after pumping for 3 times to extract 4.5 mL of super-dry tetrahydrofuran solution into the two flasks L, so that the triethylene glycol monomethyl ether fully dissolved, and then in the ice water bath environment with a syringe to extract the solution in the two flasks L into the two flasks K slowly drop, you can observe bubbles, when no bubbles removed the ice water bath, at room temperature conditions for 60 minutes reaction; subsequently, the 1.05 g of intermediate 2,5-dibromo-3-methylenebromothiophene S5 (1.2 eq, 6.456 mmol) was weighed in a 25 mL two-necked flask M and dissolved with 3.5 mL of super-dry tetrahydrofuran solution after three repetitions of gas exchange, and then slowly injected into the two-necked flask K with a syringe and reacted overnight under ambient conditions at room temperature. After the reaction, the material in the two flasks K was filtered with diatomaceous earth, accompanied by several washes of dichloromethane solution, and the filtrate was distilled under reduced pressure and then purified by silica gel column chromatography, in which the eluent was dichloromethane : ethyl acetate = 3:2. After the above operation, monomer S6 was obtained: 1.78 g of pale yellow transparent oily liquid in 84% yield. ^{1}HNMR(500MHz,C_2D_6OS) δ/ppm 7.00(s，1H), 4.44(s，2H), 3.67-3.63(m，8H),3.62-3.60(m，2H）,3.58-3.52(m，2H),3.38(s，3H).

Synthesis of polymer P3(TEG)T with different molecular weights

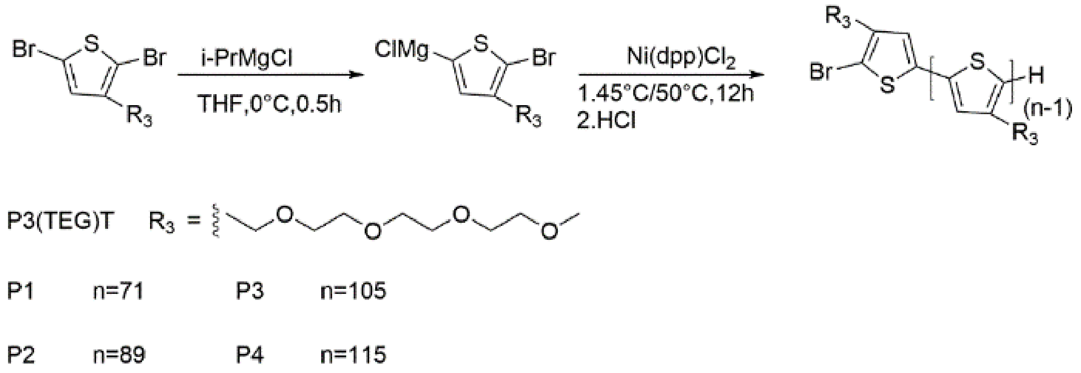

Figure S2 Synthetic route of polymer P3(TEG)T

As shown in Figure S2, the synthesis of poly(3-alkoxythiophene) P3 (TEG)T involved catalytic transfer polycondensation (CTP) reactions. The monomer-to-catalyst ratios for the polymers P1-P4 (dark red elastomers) were 90:1, 110:1, 130:1, and 150:1, respectively. Taking the example of the synthesis of poly(3-alkoxythiophene) P3 (TEG)T with a monomer-to-catalyst equivalent ratio of 90:1, the specific procedures are detailed below:

Poly(3-alkoxythiophene) P3 (TEG)T synthesis:Weigh 500 mg (1 eq, 1.25 mmol) of the monomer 2,5-dibromo-3-alkoxythiophene S1 in a pre-dried 50 mL two-neck flask F under a nitrogen atmosphere. Perform three cycles of nitrogen purging and evacuate the flask while heating and stirring using a magnetic stirrer to remove moisture and oxygen.Inject 10 mL of rigorously dried tetrahydrofuran (THF) into the flask F using a syringe under a nitrogen atmosphere. Stir for 2 minutes, then inject 1.52 mL of isopropylmagnesium chloride (iPr-MgCl) (0.98 eq, 3.04 mmol) slowly into the flask F while maintaining an ice bath. Remove the ice bath and allow the reaction to proceed in the dark at room temperature for 30 minutes, observing the solution turning pale gray.In another pre-dried 50 mL two-neck flask G, weigh 9.7 mg (0.0128 mmol) of 1,3-bis(diphenylphosphino)propane nickel(II) chloride (Ni(dppp)Cl2). Purge the flask with nitrogen three times to ensure it is free of moisture and oxygen. Under a nitrogen atmosphere, inject 2 mL of rigorously dried tetrahydrofuran (THF) into flask G to create a suspension. Rapidly inject the suspension into flask F using a 2.5 mL

syringe. Heat to 45°C and stir overnight. You will observe the solution in flask F changing from pale gray to orange-red and eventually red. When illuminated with a UV lamp, an orange-red fluorescence will be visible.After the reaction, remove the oil bath, cool to room temperature, and quench the reaction by adding a pre-prepared 1 M hydrochloric acid aqueous solution. Perform extraction using dichloromethane (or chloroform) and deionized water. Dry the organic phase over anhydrous MgSO4, filter, and obtain the crude product through vacuum distillation. Add a small amount of tetrahydrofuran (THF) (or chloroform) solution to dissolve the crude product obtained without solvents. Dropwise add this solution into a methanol solution in a centrifuge tube to precipitate the polymer. Subsequently, centrifuge the tube multiple times (at 5800 rpm for 10 minutes) to remove low molecular weight impurities. Discard the upper layer solution and place the sample in a 40°C vacuum oven for vacuum drying. After 12 hours of drying, obtain 200 mg of dark red polymer (in fluid state) with a yield of 64.7%. The room temperature state diagram of the polymer is shown in Figure S3.

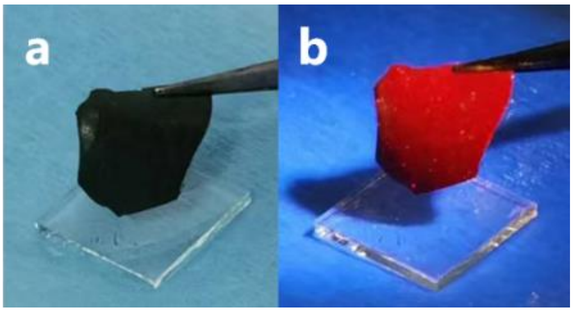

Figure S3 The state of polymer P3(TEG)T (a) ; P3(TEG)T under ultraviolet lamp (b)

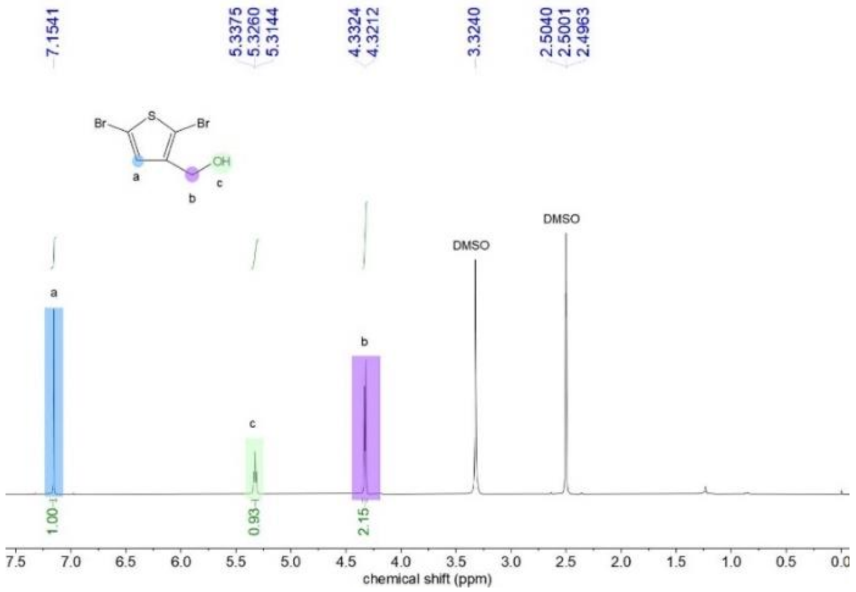

Figure S4 ^{1}H NMR spectrum of compound S4

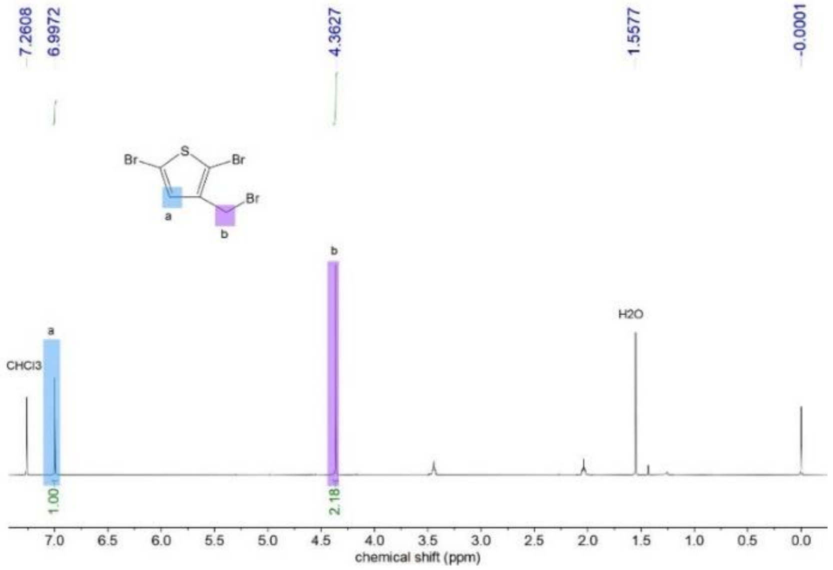

Figure S5 ^{1}H NMR spectrum of compound S5

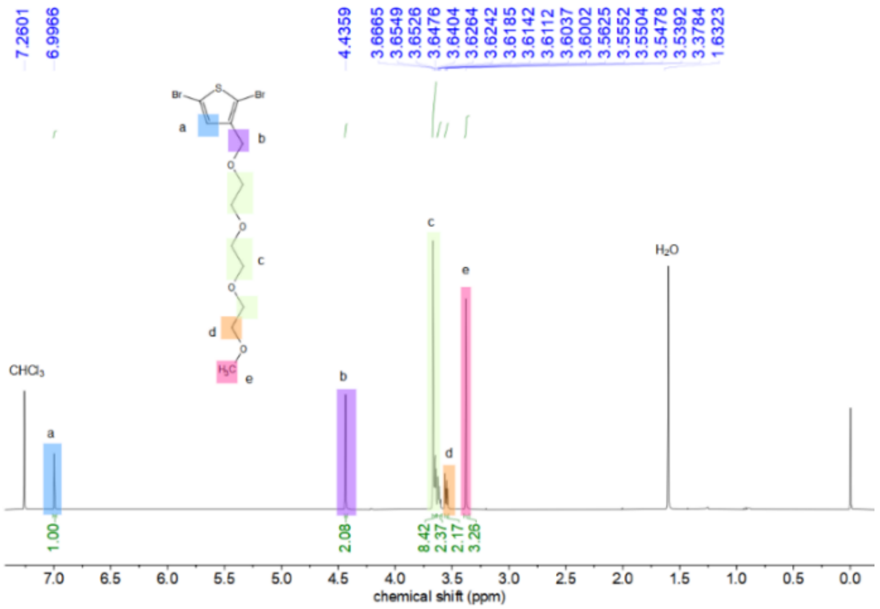

Figure S6 ^{1}H NMR spectrum of monomer S6

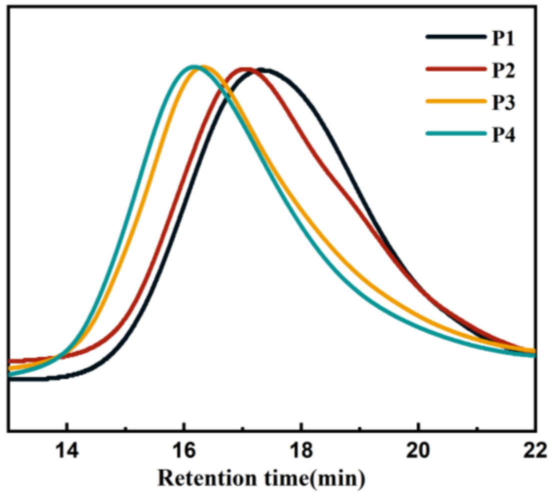

Figure S7 GPC curves of polymer P1-P4

Table S1. Summary of thermoelectric parameters of reported poly(thiophene)s/SWCNTs nanocomposites.

Nanocomposite	Casting method	σ [S cm^{-1}]	S [μV K^{-1}]	PF [μW m^{-1} K^{-2}]	Ref.
P3HT/SWCNTs	drop-casting	275	32	28	1

P3HT/SWCNTs	bar-coating	501	37.5	71.8	2
P3HT/SWCNTs	bar-coating	141	56.1	44.4	3
P3HT /SWCNTs	mold	130.8	38.7	19.6	4
P3HT /SWCNTs	drop-casting	699.6	43	65	5
PMEET /SWCNTs	drop-casting	219.1	58	121	6
P3HT/SWCNTs	drop-casting	506.9	61.2	190.6	7
P3HT/SWCNTs	Spin-coating	396.8	68.1	172.2	8
P3EHT /SWCNTs	Spin-coating	352.6	69.5	170.3	8
P3EHTT /SWCNTs	Spin-coating	486.5	65.2	202.7	8
P3HDTT /SWCNTs	Spin-coating	745.5	68.0	307.7	8
P3(TEG)T /SWCNTs(9:1)	drop-casting	24.11973	62.3159	9.15774	This work
P3(TEG)T /SWCNTs(7:3)	drop-casting	386.50841	56.29123	122.88883	This work
P3(TEG)T /SWCNTs(5:5)	drop-casting	600.40595	49.6895	147.51261	This work
P3(TEG)T /SWCNTs(3:7)	drop-casting	1115.32591	51.93027	301.66936	This work
P3(TEG)T /SWCNTs(1:9)	drop-casting	1170.90468	62.0077	449.49762	This work
P1/SWCNTs (5:5)	drop-casting	437.68608	53.61337	126.07224	This work
P2/SWCNTs (5:5)	drop-casting	349.06066	53.80037	101.56201	This work

P3/SWCNTs (5:5)	drop-casting	412.14843	55.55713	127.79265	This work
P4/SWCNTs (5:5)	drop-casting	600.40595	49.6895	147.51261	This work
P4/SWCNTs (7:3)	drop-casting	236.35018	60.56373	86.60865	This work
P4/SWCNTs (7:3)	drop-casting	344.77042	57.64083	114.56859	This work
P4/SWCNTs (7:3)	drop-casting	455.62601	48.84073	106.8053	This work
P4/SWCNTs (7:3)	drop-casting	386.50841	56.29123	122.88883	This work
P4/SWCNTs (3:7)	drop-casting	1076.03056	50.63053	263.89719	This work
P4/SWCNTs (3:7)	drop-casting	927.12335	53.07523	263.89719	This work
P4/SWCNTs (3:7)	drop-casting	795.36389	55.87783	250.005	This work
P4/SWCNTs (3:7)	drop-casting	1115.32591	51.93027	301.66936	This work

REFERENCE

1. Bounioux, C.; Díaz-Chao, P.; Campoy-Quiles, M.; Martín-González, M. S.; Goñi, A. R.; Yerushalmi-Rozen, R.; Müller, C., Thermoelectric Composites of Poly(3-Hexylthiophene) and Carbon Nanotubes with a Large Power Factor. *Energy Environ. Sci.* **2013,** *6* (3), 918.
2. Lee, W.; Hong, C. T.; Kwon, O. H.; Yoo, Y.; Kang, Y. H.; Lee, J. Y.; Cho, S. Y.; Jang, K. S., Enhanced Thermoelectric Performance of Bar-Coated Swcnt/P3ht Thin Films. *ACS Appl. Mater. Interfaces* **2015,** *7* (12), 6550-6.
3. Hong, C. T.; Lee, W.; Kang, Y. H.; Yoo, Y.; Ryu, J.; Cho, S. Y.; Jang, K.-S., Effective Doping by Spin-Coating and Enhanced Thermoelectric Power Factors in Swcnt/P3ht Hybrid Films. *J. Mater. Chem. A* **2015,** *3* (23), 12314-12319.
4. Kang, Y. H.; Lee, U.-H.; Jung, I. H.; Yoon, S. C.; Cho, S. Y., Enhanced Thermoelectric Performance of Conjugated Polymer/Cnt Nanocomposites by Modulating the Potential Barrier Difference between Conjugated Polymer and Cnt. *ACS Appl. Electron. Mater.* **2019,** *1* (7), 1282-1289.
5. Hao, L.; Kang, J.; Shi, J.; Xu, J.; Cao, J.; Wang, L.; Liu, Y.; Pan, C., Enhanced Thermoelectric Performance of Poly(3-Substituted Thiophene)/Single-Walled Carbon Nanotube Composites Via Polar Side Chain Modification. *Compos. Sci. Technol.* **2020,** *199*, 108359.
6. Liu, C.; Yin, X.; Chen, Z.; Gao, C.; Wang, L., Improving the Thermoelectric Performance of Solution-Processed Polymer Nanocomposites by Introducing Platinum Acetylides with Tailored Intermolecular Interactions. *Chem. Eng. J.* **2021,** *419*.
7. Zhou, X.; Pan, C.; Liang, A.; Wang, L.; Wong, W.-Y., Thermoelectric Properties of Composite Films Prepared with Benzodithiophene Derivatives and Carbon Nanotubes. *Compos. Sci. Technol.* **2017,** *145*, 40-45.

8. Lin, P.-S.; Inagaki, S.; Liu, J.-H.; Chen, M.-C.; Higashihara, T.; Liu, C.-L. The Role of Branched Alkylthio Side Chain on Dispersion and Thermoelectric Properties of Regioregular Polythiophene/Carbon Nanotubes Nanocomposites. *Chem. Eng. J.* 2023, 458, 141366.
